# Effects of 4 weeks of β-alanine supplementation on aerobic fitness in water polo players

**DOI:** 10.1371/journal.pone.0205129

**Published:** 2018-10-11

**Authors:** Gabriel Motta Pinheiro Brisola, Paulo Eduardo Redkva, Dalton Muller Pessôa Filho, Marcelo Papoti, Alessandro Moura Zagatto

**Affiliations:** 1 Post-Graduate Program in Movement Sciences, Sao Paulo State University—UNESP, Bauru, Brazil; 2 Laboratory of Physiology and Sport Performance (LAFIDE)—Sao Paulo State University—UNESP, School of Sciences, Department of Physical Education, Bauru, Brazil; 3 Sao Paulo State University—UNESP, School of Sciences, Department of Physical Education, Bauru, Brazil; 4 School of Physical Education and Sport of Ribeirão Preto, University of Sao Paulo, Ribeirão Preto, Brazil; East Stroudsburg University, UNITED STATES

## Abstract

The purpose of the present study was to investigate the ergogenic effects of 4 weeks of β-alanine supplementation on peak oxygen uptake ($\dot{V}O_{2\mathrm{peak}}$) and force associated with $\dot{V}O_{2\mathrm{peak}}$ ($F\dot{V}O_{2\mathrm{peak}}$) during a tethered swimming graded exercise test, and a three-minute all-out effort (3min_ALL-OUT_) in water polo players. Twenty-two male national competitive level water polo players were randomly assigned to receive either 4 weeks of β-alanine (n = 11) or a placebo (n = 11) (i.e., 4.8 g·day^-1^ for 10 days, then6.4 g·day^-1^ for 18 days, resulting in 163.2 g over 28 days). The participants performed the TS_GET_ and 3min_ALL-OUT_ before and after the supplementation period. There were no significant interaction effects between-groups for any variable, however, the magnitude-based inferences analyses showed a *possibly beneficial* effect (74%) of β-alanine supplementation on $F\dot{V}O_{2\mathrm{peak}}$ compared to placebo treatment (Δ% [post–pre] for Placebo group = -5.2%; Δ% [post–pre] for β-alanine group = +0.7%). Only the β-alanine group presented a significant reduction in $\dot{V}O_{2\mathrm{peak}}$ expressed in absolute values (PRE = 3.3±0.6L·min^-1^; POST *=* 3.0±0.4L·min^-1^; *p* = .021). Similarly, only the β-alanine group presented a significant increase in critical force (PRE = 51.2±10.4N; POST = 56.5±13.1N; *p* = .044) and a reduction in the curvature constant parameter (W'; PRE = 2998.0±1103.7N·s; POST = 2224.6±1058.9N·s; *p =* .049). Thus, we can conclude that 4 weeks of β-alanine supplementation presented mixed results in water polo players, indicating that this nutritional strategy may not be effective in improving parameters of the TS_GET_ and 3min_ALL-OUT ._

## Introduction

Water polo is a high-intensity intermittent team sport [1,2] that presents alternated aerobic-anaerobic metabolic demand [3]. The repeated high-intensity efforts in water polo, which occur several times (6.7±3.5 bouts of repeated high-intensity efforts composed of 4.6 efforts each) during a match [2], are considered an important factor for the success of the game [2]. In fact, a muscle recovery process between efforts, such as phosphocreatine replenishment and the removal of accumulated intracellular inorganic phosphates, which are oxygen-dependent processes [4], is crucial for performance during repeated high-intensity efforts [4]. Therefore, aerobic fitness is also important in water polo, evidenced through the high levels of peak oxygen uptake ($\dot{V}O_{2\mathrm{peak}}$) found in elite athletes, the top four in the World Championship (5.2 ± 0.4 L·min^-1^), and high swimming velocity corresponding to anaerobic threshold (1.33 ± 0.05 m∙s^-1^) [5].

Due to the importance of repeated efforts in water polo, nutritional strategies to improve repeated effort ability have been a focus of some studies [6–8], especially ergogenic aids that could improve the buffering capacity [6–8], since repeated efforts can promote increases in muscular acidosis [9], which is considered an important factor to promote fatigue [10]. Initially, sodium bicarbonate supplementation was investigated [6], however no beneficial effects were found, while more recently, some studies investigated the effects of β-alanine supplementation [7,8], showing a small but substantial beneficial effect on performance [7,8].

β-alanine is a non-essential amino acid and its availability is considered the limiting factor for intramuscular synthesis of carnosine [11], a cytoplasmic dipeptide (synthesized by the carnosine synthetase enzyme) which can increase >40% after 4 weeks of β-alanine supplementation in a protocol using daily doses of 1.6–6.4 g∙day^-1^ [12,13]. Carnosine (β-alanyl-L-histidine) has multiple functions [e.g., antioxidant [14], anti-glycation of proteins [15], and muscle contractility regulation [16], of which the main function is as an intracellular acid buffer [12] due to a pKa of 6.83 [17]. Thus, 4 weeks of β-alanine supplementation is enough to improve muscle buffering capacity by ~4% [11,12], which promotes high accumulations of ions H^+^ in muscle [10], and consequently improves high-intensity efforts that last between 1 and 4 min [10,18], such as repeated sprints in water polo [7,8] and total work done at 110% in cycling [12], among others.

In the way, some studies also have reported that β-alanine supplementation can improve the time-to-exhaustion during a maximal graded test [19,20], distance covered during the Yo-Yo Intermittent Recovery Test Level 2 [21], and even $\dot{V}O_{2\mathrm{peak}}$ [20,22]. The possible mechanism for improvement in the aerobic fitness index is that β-alanine supplementation could shift the lactate curve to the right [19,23], delaying fatigue establishment, probably reducing muscle acidosis during exercise, which can improve the time-to-exhaustion, and reducing the enzymatic inhibition [24,25] allowing greater activity of the oxidative metabolism and possibly improving some aerobic parameters, as shown by some studies [19,21, 18, 22].

Thus, due to the fact that aerobic fitness is important to water polo, β-alanine supplementation may be appropriate for water polo players. In an isolated study in water polo for aerobic outcomes, Brisola et al. [8] reported that maximal distance covered during a 30-minute swimming test (which has a strong relationship with the anaerobic threshold [r = 0.90] [26] did not improve after 4 weeks of β-alanine supplementation, however this effort was performed immediately after a repeated sprint ability test in an attempt to mimic the physical stress during a game, instead of a study designed to investigate the effects of β-alanine supplementation on aerobic parameters. Thereby, the ergogenic effects of β-alanine supplementation on aerobic parameters require further investigation, since there are other studies that did not find improvement in these parameters [23,27–29].

Therefore, the aim of the present study was to investigate the effects of 4 weeks of β-alanine supplementation on $\dot{V}O_{2\mathrm{peak}}$ and force associated with $\dot{V}O_{2\mathrm{peak}}$ ($F\dot{V}O_{2\mathrm{peak}}$) during a tethered swimming graded exercise test (TS_GET_), and a three-minute all-out effort (3min_ALL-OUT_) in water polo players. We hypothesized that β-alanine supplementation would improve, especially, $F\dot{V}O_{2\mathrm{peak}}$ in TS_GET_ and parameters related to endurance capacity of 3min_ALL-OUT,_ through improvement in the time to exhaustion due to delay in acidosis during these tests. Furthermore, we also expected that β-alanine supplementation would improve anaerobic fitness.

## Materials and methods

### Subjects

Twenty-two male national competitive water polo athletes participated in the study (mean±SD: age = 18±4 yrs, body weight = 78.5±9.5 kg and height = 1.79±0.06 m), separated into two homogenous groups: placebo (n = 11; age: 18±3 yrs, body mass: 81.4±8.8 kg, height: 1.79±0.07 m; protein intake: 2.0±0.6 g·kg^-1^·day^-1^ and player positions: 4 goalkeepers; 1 center forward; 2 central defenders, and 4 field players) and β-alanine (n = 11; age: 19±5 yrs, body mass: 75.7±9.6 kg, height: 1.79±0.06 m; protein intake: 2.0±0.5 g·kg^-1^·day^-1^ and player positions: 2 goalkeepers, 1 center forward, 2 central defenders, and 6 field players). The participants were required to have at least 3 years of competitive experience. The team to which the athletes belonged won the elite national water polo championship in the year the study was conducted. When the study began, the players were in the general preparatory phase of the season, while at the end of the study, they were in a competitive phase. The training routine was composed of two training sessions per day and the weekly training frequency was ~5 days a week (~10 sessions a week). The training load was monitored during the study period using the session rating of perceived exertion method [30] (i.e., multiplying the rating of perceived exertion by total session duration in minutes) and the weekly values of total training load were: first week = 3,723±902 arbitrary units, second week = 3,764±843 arbitrary units, third week = 4,494±1,223 arbitrary units, and fourth week = 4,522±1,620 arbitrary units. The daily training sessions were not influenced by the researchers.

Participants were instructed not to take any dietary supplements for 3 months prior to the start of the study. Furthermore, during the study participants were not permitted to take any dietary supplement that was not provided by the researchers (i.e., placebo or β-alanine) and were instructed to maintain their usual diets. Participants who were repeatedly absent during training or assessment, were injured, or dropped-out of the team, were excluded from the study (5 participants were excluded). The participants were informed about the possible risks and benefits of the study prior to signing an informed consent, and all procedures were conducted in accordance with the Declaration of Helsinki. In case of participants under 18 years of age, the informed consent was signed by their parents. The experimental procedures used in the study, as well as the informed consent, were approved by the Research Ethics Committee of the Sao Paulo State University-UNESP (Protocol number 430.916/2013).

### Experimental design

The study was conducted in a double-blind, randomized, parallel, and placebo-controlled manner.

Initially, the participants performed the (TS_GET_, a specific test used for determination of $\dot{V}O_{2\mathrm{peak}}$) and ($F\dot{V}O_{2\mathrm{peak}}$). Twenty-four hours later, a 3min_ALL-OUT_ was performed principally for determination of critical force and the curvature constant parameter (W'), also in a tethered swimming exercise, which correspond to endurance capacity and anaerobic work capacity, respectively. These tests were part of a larger testing battery using the same participants [8]. In the following week, the participants were randomly allocated to receive either a placebo or β-alanine for 4 weeks. Randomization was equalized by athlete position and protein intake (measured by a 3-day food recall) to ensure homogeneity between groups. After the supplementation period (one day after the last dose ingested), all participants were reassessed.

### Experimental procedures

The assessments and reassessments were performed in an indoor swimming pool at 27±1°C.

The TS_GET_ and 3min_ALL-OUT_ were performed in tethered front crawl style for measurement of force. The participants were attached by a belt to an elastic cord (Auriflex n-204, São Roque, São Paulo, Brazil) connected to a system for measurement of force, which was positioned on the edge of the pool. The force measurement system used was a high frequency signal acquisition (1000 Hz), comprising a load cell (CSA/ZL-100 kg, MK Controle, São Paulo, SP, Brazil) fixed to the starting blocks, a signal conditioner (MK Controle, São Paulo, SP, Brazil), an analog/digital signal acquisition module (USB-6008, National Instruments, Austin, TX, USA), and appropriate software (LabView 9—National Instruments, Signal Express, Austin, TX, USA). Posteriorly, the acquired data was filtered by a 4^th^ order Butterworth filter with a 10Hz cutoff frequency in MatLab^®^ software (MatLab, MathWorks, Natick, MA, USA).

From their training sessions, the participants were already familiar with tethered swimming efforts and swimming with the snorkel (AquaTrainer^®^, Cosmed, Rome, Italy) used for measurement of oxygen uptake ($\dot{V}O_{2}$) in the TS_GET_. Furthermore, during the specific in-water warm-up (about 5 min) performed before the tests, the participants were allowed to use tethered swimming system in the case that they did not yet feel familiar. In addition, a warm-up was also performed on dry-land (static and dynamic stretching) for about 5 min.

During the TS_GET_, the $\dot{V}O_{2}$ was measured breath-by-breath by a portable gas analyzer (K4b2, Cosmed, Rome, Rome, Italy) which was calibrated after 4 tests (tests were performed in sequence and therefore, calibrated approximately once every 160 min) with ambient air samples and gas of known constituents (CO_2_ = 3.98% and O_2_ = 16.02%). For posterior analyses, the $\dot{V}O_{2}$ data were subsequently smoothed every 30 points to exclude outlier points [31].

In both tests, capillary blood (25μL) was sampled from the earlobe 3, 5, and 7 minutes after the test for analysis of peak blood lactate ([La^-^]_peak_). Capillary blood (25µL) was also collected at rest (participants remained seated for 10 min) before the start of the 3min_ALL-OUT_ ([La^-^]_resting_)_._ The samples were collected in heparinized capillary tubes and stored at -20ºC until analysis in microtubes containing 50 µL of 1% sodium fluoride. Subsequently (one month after), the blood samples were analyzed in an electrochemical lactate analyzer YSI 2300 (YSI, Yellow Spring Instruments, Yellow Springs, Ohio, USA) (measurement error±2%).

#### Tethered swimming graded exercise test (TS_GET_)

**Fig 1:**
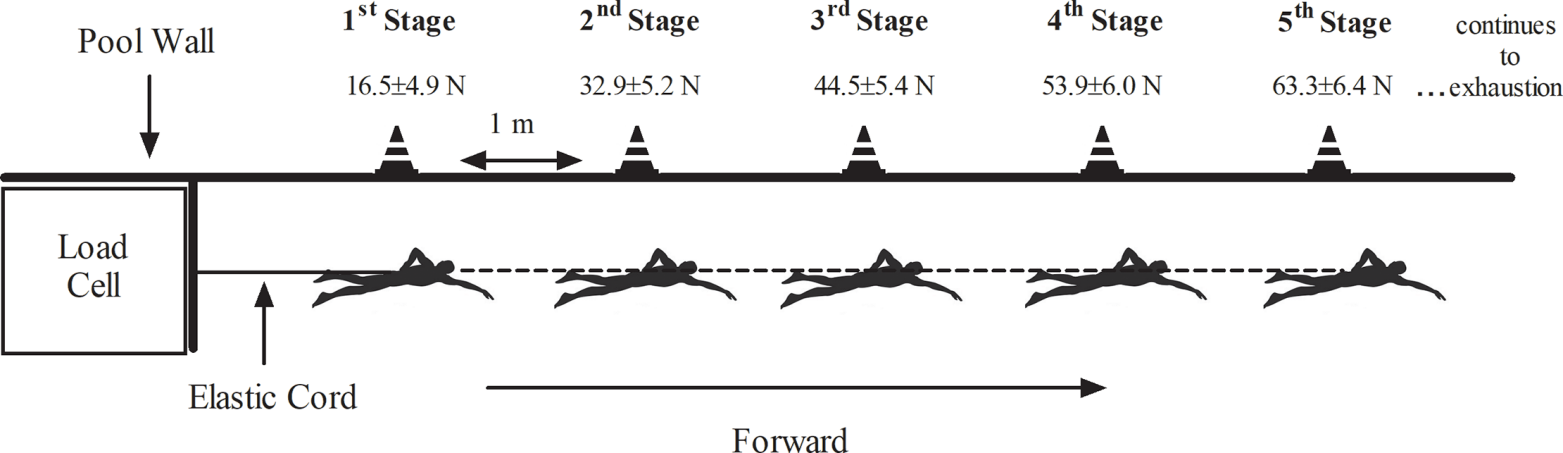
Tethered swimming graded exercise test. A diagram of the cone positions at the side of the pool used as reference points for the intensities during the tethered swimming graded exercise test (TS_GET_). Adapted from Papoti et al. [32].

The TS_GET_ was adapted from Papoti et al. [32] (Fig 1), which presented a significant relationship with free swimming performance [32] and sensitivity to training responses [33]. The TS_GET_ is a maximum specific test used for determination of $\dot{V}O_{2\mathrm{peak}}$ (which is considered the best predictor of cardiovascular fitness [34]) and $F\dot{V}O_{2\mathrm{peak}}$.

The participants were attached to a high-frequency signal acquisition system (as described in the experimental procedures) by an elastic cord, 3 meters long, and were required to displace the cord forward to progress swimming-workload for each stage until exhaustion. Each exercise stage of the TS_GET_ was signaled by cones on the edge of the pool, and the increase in exercise workload was performed by increasing the tension on the elastic cord when the participants advanced stages. The workload for the first stage corresponded to 16.5±4.9 N, increasing 11.5±1.1 N every minute to the next stage.

During each stage, the participants were instructed to keep their waistline as close as possible to the level of the cones, which were positioned on the edge of the pool every 1 m. Exhaustion was considered as the impossibility of participants to maintain their waist aligned with the cone (i.e., ≥ 5 s swimming out of stage) or by volitional exhaustion (i.e., participants gave up continuing the test).

The workload for each stage was considered as the average force recorded during the final 50 seconds of each stage. The $\dot{V}O_{2\mathrm{peak}}$ was taken as the highest average found in the test (considering only the final 20 s), while the $F\dot{V}O_{2\mathrm{peak}}$ was equivalent to the lowest workload (force) where the $\dot{V}O_{2\mathrm{peak}}$ was attained [34]. If this stage was not completed, the force associated with this stage was determined using the formula of Kuipers et al. [35].

$$F\dot{V}O_{2\mathrm{peak}}=F+((a\times n)/b)$$ (1)

where “F” is the force (N) of the final completed stage, “a” is the increment in the test (N), “n” is the duration (s) maintained in the incomplete stage, and “b” is the duration (s) of the stage.

#### Three-minute all-out effort (3min_ALL-OUT_)

The 3min_ALL-OUT_ was performed as described by Kalva-Filho et al. [36], which presented a significant relationship with free swimming performance and the physiological index [36,37].

The 3min_ALL-OUT_ was composed of a maximum effort of three minutes duration in tethered swimming, where athletes were attached to a double elastic cord of 3 m and instructed to perform their maximum effort for 3 minutes without any strategy and without receiving feedback time. The beginning and end of the test were always marked by a loud whistle.

The measurement of force was performed during the 3min_ALL-OUT_ principally to determine critical force and the W'. The critical force was the corresponding mean value obtained in the final 30 seconds of the all-out trial and is an alternative method for determining the upper-limit heavy exercise domain (aerobic parameter) [38]. The W' was assumed to be the area under the slope for the relationship between force and time located above critical force, which is often referred to as an index related to anaerobic work capacity [38].

In addition, the following parameters were determined: peak force, mean force, integral of force (area under the curve), relative fatigue index (Eq 2), and absolute fatigue index (Eq 3) during swimming.

relativefatigueindex=(peakforce‑minimumforce)×100/peakforce (2)

absolutefatigueindex=(peakforce×testtime)‑integralofforce (3)

#### Supplementation protocol

Participants received either β-alanine (99.9% pure β-alanine; CarnoSyn, NAI, San Marcos, California, USA) or a placebo (dextrose, Neonutri, Poços de Caldas, Minas Gerais, Brazil) for 4 weeks. Both the placebo and β-alanine were powdered and packed in gastro-resistant capsules (hidroxipropilmetilcelulose, DrCaps, Capsugel, Puebla, Puebla, Mexico), identical in appearance (i.e., white, opaque, and size 00), which were produced in the laboratory using a manual capsule filling machine (Ideal, Arujá, Sao Paulo, Brazil). The supplements were delivered as weekly doses. On the first 10 days, participants received 4.8 g·day^-1^, divided into six daily doses of 800 mg and on the final 18 days, participants received doses corresponding to 6.4 g·day^-1^, divided into four daily doses of 1600 mg. All doses were taken with an interval of at least two hours between them and after meals to avoid the occurrence of paresthesia [12]. If participants forgot to ingest the capsules, they were instructed to ingest them on the following days, maintaining the pattern of ingestion. The participants were individually monitored during the study regarding side-effects of supplementation (i.e., paresthesia) and were instructed not to comment about side-effects to other players.

#### Statistical analysis

The data are presented as means±SD and confidence interval of 95% (95%CI). To compare the outcomes, between-subject factor (placebo vs. β-alanine) and within-subject factor 'time' (pre vs. post), a two-way repeated measure ANOVA was used for all variables. In addition, for analysis of variance, the Mauchly sphericity test was applied and sphericity was assumed when no significant F value was observed. In case of violation of sphericity, the Epsilon Greenhouse-Geisser correction was used. The analyses were completed with the Bonferroni *post hoc*. The unpaired t-test was used to compare the percentage change in score (Δ%; i.e., values post supplementation minus values pre-supplementation), between-groups. In all cases, a 5% significance level (*p*≤0.05) was adopted and the data were analyzed in SPSS version 15.0 for Windows (SPSS Inc., Chicago, IL, USA).

In addition to conventional statistical analysis, magnitude-based inference analysis was used for comparisons between-subject factor (placebo vs. β-alanine) [39]. The values are expressed as the standardized mean difference (Cohen's *d*±Confidence Limits of 90%) [40], which was calculated using a pooled standard deviation as the denominator. The threshold values for Cohen’s *d* statistical power were considered as > 0.2 (small), >0.5 (moderate), and >0.8 (large), and the chances of the effect being positive, trivial, or negative were calculated based on the smallest worthwhile change (SWC = 0.2 x SD). If the probabilities of the effect being substantially positive and negative were both > 5%, the effect was reported as unclear, or, if not, the effect was clear. Thus, the changes were qualitatively evaluated as follows: <1% *= most unlikely*; 1%–5% = *very unlikely*; 5%–25% = *unlikely*; 25%–75% = *possibly*; 75%–95% = *likely*; 95%–99% = *very likely*; and >99% = *most likely* [41].

## Results

In total 4 participants reported paresthesia during the study, of which 3 were in the β-alanine group and 1 in the placebo group (note: all were maintained in the statistical analysis).

There were no significant interaction effects between-groups for any variable.

The results of the TS_GET_ are shown in Table 1. The magnitude-based inference analysis showed a *possibly beneficial* effect on $\dot{\mathrm{FV}}O_{2\mathrm{peak}}$ for β-alanine supplementation compared to the placebo. There was a significant difference within-group (pre vs. post) only for the β-alanine group in $\dot{V}O_{2\mathrm{peak}}$ expressed in absolute values (Statistical Power = 85%; F = 10.2; *p* = 0.005; *post hoc p* = 0.021) (S1 File).

**Table 1:** Tethered swimming graded exercise test parameters pre and post supplementation.

	Placebo Group			β-alanine Group			Magnitude-based Inference Analysis (β-alanine Group × Placebo Group)		
	Pre	Post	Δ%	Pre	Post	Δ%	Effect size (Cohens’s*d* ± 90% CL)	%Chances (beneficial/ trivial/ harmful)	Qualitative inference
$\dot{V}O_{2\mathrm{peak}}$ **(L·min**^**-1**^**)**	3.4±0.6 (3.1 to 3.7)	3.2±0.5 (2.9 to 3.4)	-8.2	3.3±0.6 (3.0 to 3.6)	3.0±0.4* (2.7 to 3.2)	-9.8	-0.1±0.6	20/40/40	*unclear*
$\dot{V}O_{2\mathrm{peak}}$ **(mL·kg**^**-1**^**·min**^**-1**^**)**	41.7±8.0 (37.5 to 46.4)	40.1±8.9 (35.4 to 44.9)	-6.9	43.4±4.9 (40.4 to 45.9)	39.9±7.9 (35.8 to 44.6)	-8.0	0.0±0.7	28/38/34	*unclear*
**$F\dot{V}O_{2\mathrm{peak}}$ (N)**	81.7±15.4 (73.5 to 90.8)	77.6±10.0 (72.2 to 83.2)	-5.2	75.6±12.1 (68.5 to 81.9)	76.3±18.0 (66.4 to 85.8)	0.7	0.4±0.6	74/21/04	*possibly beneficial*
**TTE (s)**	429.0±102.3 (367.3 to 486.4)	424.5±104.6 (360.0 to 482.1)	-1.1	413.8±90.9 (362.5 to 462.1)	424.3±116.0 (358.8 to 492.4)	3.5	0.1±0.5	42/46/12	*unclear*
**[La**^**-**^**]**_**peak**_ **(mmol·L**^**-1**^**)**	7.7±2.5 (6.4 to 9.1)	6.6±1.7 (5.7 to 7.5)	-8.2	8.5±2.5 (6.6 to 9.5)	7.3±2.1 (6.2 to 8.5)	-12.4	0.1±0.7	44/36/20	*unclear*

Values expressed as Mean±SD (95%CI). $F\dot{V}O_{2\mathrm{peak}}$ = force associated with peak oxygen uptake; TTE = time to exhaustion; $\dot{V}O_{2\mathrm{peak}}$ = peak oxygen uptake; [La^-^]_peak_ **=** peak blood lactate. Δ % = percentage difference (post–pre). The quantitative chances were assessed qualitatively as follows: <1% *= most unlikely*; 1%–5% = *very unlikely*; 5%–25% = *unlikely*; 25%–75% = *possibly*; 75%–95% = *likely*; 95%–99% = *very likely*; and >99% = *most likely*. If the probabilities of the effect being substantially beneficial and harmful were both > 5%, the effect was reported as *unclear*.

The magnitude-based inference analysis presents the percentage chance or likelihood that the true effect of the β-alanine supplementation will be beneficial, trivial, or harmful when compared with the placebo condition.

*Significant difference from pre-supplementation (*p*≤0.05).

The results of the 3min_ALL-OUT_ are shown in Table 2. There was a significant improvement within-group only in the β-alanine group for critical force (Statistical Power = 65%; F = 6.0; *p* = 0.024; *post hoc p* = 0.044), while the W' dropped significantly (Statistical Power = 56%; F = 4.9; *p* = 0.039; *post hoc p = 0*.049) (S1 File). The magnitude-based inference analysis showed an *unclear* effect for all variables of the 3min_ALL-OUT._

**Table 2:** The three-minute all-out effort parameters pre and post supplementation.

	Placebo Group			β-alanine Group			Magnitude-based Inference Analysis (β-alanine Group × Placebo Group)		
	Pre	Post	Δ%	Pre	Post	Δ%	Effect size (Cohens’s*d*± 90%CL)	%Chances (beneficial/ trivial/ harmful)	Qualitative inference
**Peak force (N)**	139.8 ±26.6 (126.1 to156.8)	143.4±23.0 (130.3 to 155.8)	3.9	134.4±17.9 (124.0 to 143.8)	136.5±20.9 (124.8 to 148.5)	2.0	-0.1±0.6	23/42/35	*unclear*
**Mean force (N)**	70.8±10.7 (65.4 to 77.2)	72.7±13.2 (66.1 to 80.6)	2.4	67.9±10.3 (62.5 to 73.3)	68.9±10.6 (62.9 to 74.7)	1.7	-0.1±0.3	06/68/26	*unclear*
**Fatigue index (N·s)**	12433.5±3500.2 (10472.4 to 14644.2)	12706.1±3271.8 (10816.1 to 14462.4)	8.8	11921.5±3009.4 (10141.3 to 13546.3)	12154.8±3898.1 (9916.8 to 14322.4)	2.8	0.0±0.8	34/33/33	*unclear*
**Fatigue index (%)**	71.7±9.7 (66.3 to 77.4)	67.7±7.4 (63.3 to 71.8)	-4.1	71.4±8.9 (66.5 to 76.0)	72.3±12.1 (65.7 to 79.3)	1.6	0.5±0.8	08/19/73	*unclear*
**Integral (N·s)**	12765.9±1941.2 (11785.2 to 13909.5)	13087.4±2402.3 (11889.5 to 14516.2)	2.2	12175.1±1859.1 (11180.3 to 13168.2)	12387.0±1898.4 (11315.4 to 13426.6)	2.1	-0.1±0.3	09/69/22	*unclear*
**Critical force (N)**	56.3±12.3 (49.9 to 63.6)	59.6±12.0 (53.7 to 66.7)	7.1	51.2±10.4 (45.7 to 57.0)	56.5±13.1* (49.4 to 63.7)	11.2	0.2±0.5	45/44/11	*unclear*
**W' (N·s)**	2891.8±753.0 (2477.6 to 3351.3)	2359.5±855.8 (1888.3 to 2821.4)	-8.4	2998.0±1103.7 (2410.3 to 3606.7)	2224.6±1058.9* (1675.2 to 2826.8)	-17.1	-0.4±0.9	15/23/62	*unclear*
**[La**^**-**^**]**_**resting**_ **(mmol·L**^**-1**^**)**	1.2±0.4 (1.0 to 1.4)	1.2±0.2 (1.1 to 1.3)	6.6	1.2±0.3 (1.0 to 1.3)	1.3±0.4 (1.1 to 1.5)	22.1	0.4±0.8	66/24/10	*unclear*
**[La**^**-**^**]**_**peak**_ **(mmol·L**^**-1**^**)**	13.6±2.9 (11.8 to 15.4)	12.5±3.4 (10.7 to 14.8)	-6.5	13.6±3.1 (11.9 to 15.5)	12.0±2.6 (10.6 to 13.5)	-10.1	-0.2±0.7	16/34/49	*unclear*

Values expressed as Mean±SD (95%CI). [La^-^]_peak_ **=** peak blood lactate; [La^-^]_resting_ **=** resting blood lactate; W' = curvature constant parameter. Δ % = percentage difference (post–pre). The quantitative chances were assessed qualitatively as follows: <1% *= most unlikely*; 1%–5% = *very unlikely*; 5%–25% = *unlikely*; 25%–75% = *possibly*; 75%–95% = *likely*; 95%–99% = *very likely*; and >99% = *most likely*. If the probabilities of the effect being substantially beneficial and harmful were both > 5%, the effect was reported as *unclear*.

The magnitude-based inference analysis presents the percentage chance or likelihood that the true effect of the β-alanine supplementation will be beneficial, trivial, or harmful when compared with the placebo condition.

*Significant difference from pre-supplementation (*p*≤0.05).

## Discussion

The present study investigated the effects of 4 weeks of β-alanine supplementation on $\dot{V}O_{2\mathrm{peak}}$ and $F\dot{V}O_{2\mathrm{peak}}$ from a TS_GET_ and a 3min_ALL-OUT_ in water polo players. The present study was the first to verify the effect of β-alanine supplementation on $\dot{V}O_{2\mathrm{peak}}$ in a specific-swimming ergometer test. The main finding of the present study was that β-alanine supplementation demonstrated a *possibly beneficial* effect on $F\dot{V}O_{2\mathrm{peak}}$ reached in the TS_GET_ and only the β-alanine group improved critical force in the 3min_ALL-OUT._ On the other hand, only the β-alanine group presented a significant reduction in $\dot{V}O_{2\mathrm{peak}}$ expressed in absolute values in the TS_GET_ and in the W' in the 3min_ALL-OUT._ Thus, 4 weeks of β-alanine supplementation presented mixed results in water polo players, with a slight enhancement in $\dot{\mathrm{FV}}O_{2\mathrm{peak}}$ in the TS_GET_ and critical force in the 3min_ALL-OUT_ and a slight decrease in $\dot{V}O_{2\mathrm{peak}}$ and W'.

The TS_GET_ was used in the present study mainly because it allows direct determination of $\dot{V}O_{2\mathrm{peak}}$ in a specific-swimming ergometer test, which is considered the best predictor of cardiovascular fitness [34] and has some importance for water polo [3]. However, the results of the present study showed no substantial effect of improvement in the β-alanine group compared to the placebo group for this variable, on the contrary, only the β-alanine group showed a significant reduction in $\dot{V}O_{2\mathrm{peak}}$ when the values were expressed in absolute units.

These findings could suggest a slight worsening in aerobic fitness with β-alanine supplementation, which could impair the recovery between repeated high-intensity efforts. However, the study of Brisola et al. [8] with water polo players showed that 4 weeks of β-alanine supplementation slightly improved repeated sprint ability in specific-protocol testing. Furthermore, some studies also did not find significant changes in $\dot{V}O_{2\mathrm{peak}}$ after supplementation with β-alanine [19,23,42], indicating that β-alanine supplementation may have little or no influence on this physiological variable. In addition, the only study [27] in the literature that found a significant reduction in $\dot{V}O_{2\mathrm{peak}}$ with β-alanine supplementation did not use pure β-alanine and the authors considered the reduction as an unexpected effect. Thus, the significant reduction in $\dot{V}O_{2\mathrm{peak}}$ found in the present study appears to be more associated with the seasonal training phase of the team, as both groups presented a reduction in Δ% (Table 1), low effect sizes (ES = -0.1±0.6) and the magnitude-based inference analyzes showed that these differences between groups are *unclear*. In a study with team sports athletes (in this case, rugby players), Taylor et al. [43] showed that maximal oxygen uptake ($\dot{V}O_{2\max}$) can be decreased after 6 weeks of in-season training. Furthermore, the study of Taylor et al. [43] also showed a curvilinear relationship between training load and changes in $\dot{V}O_{2\max}$, demonstrating that high weekly training load can decrease the $\dot{V}O_{2\max}$. Therefore, the decrease in $\dot{V}O_{2\mathrm{peak}}$ after β-alanine supplementation found in the present study should be analyzed with caution and further studies are needed to confirm if there is a possible deleterious effect of β-alanine supplementation on $\dot{V}O_{2\mathrm{peak}}$.

In addition, a small indication of a beneficial effect (74% *beneficial effect*) was found in $F\dot{V}O_{2\mathrm{peak}}$ in the TS_GET_ compared to the placebo group. Previous studies have found improvement in performance parameters with β-alanine supplementation in a maximal graded test, such as an increase in time to exhaustion on a cycle ergometer [19,20], which indicates a slightly positive effect of β-alanine supplementation on delayed acidosis and improved time to exhaustion. However, in the present study this improvement between groups shown by the magnitude-based inference analysis may be more due to deterioration in $F\dot{V}O_{2\mathrm{peak}}$ in the placebo group (-5.2%) and therefore, the supplementation of β-alanine showed a marginal effect of improvement.

The 3min_ALL-OUT_ was used in the present study as it enables assessment of aerobic, anaerobic, and high-intensity performance parameters in a specific swimming test [36]. The only improvement found with β-alanine supplementation in the 3min_ALL-OUT_was in critical force, which is a parameter representative of the aerobic metabolism (intensity of which could theoretically be endlessly maintained without the occurrence of exhaustion) [38]. The relevance of this parameter determined in the 3min_ALL-OUT_ was shown by Kalva-Filho et al. [36], who found a strong correlation between critical force (r = 0.80) and the workload of swimming corresponding to the lactate minimum. Therefore, this slight improvement in critical force could represent a slight improvement in endurance capacity of the athletes. Since water polo players cover long distances in a match (~1600 m) [1], composed of several repeated high-intensity effort bouts [2], endurance capacity could have an important role in phosphocreatine replenishment and removal of metabolites during the intermittent high-intensity efforts [4]. However, caution is required in any statement due to the mixed results found in aerobic fitness (i.e., reduction in $\dot{V}O_{2\mathrm{peak}}$). The previous study showed that β-alanine supplementation was not able to improve the endurance capacity in a 30-minute swimming test, however, this effort was performed immediately after an RSA to simulate the metabolic demand of the water polo game, instead of a study designed to investigate the effects of β-alanine supplementation on aerobic parameters. On the other hand, in studies performed on a cycle ergometer, improvements in endurance capacity such as ventilatory threshold and lactate threshold were found [19,23].

Contrary to a slight enhancement in critical force, the present study found a significant reduction in W' only for the β-alanine group in the 3min_ALL-OUT_. The W' is a mathematically derived-parameter that represents the amount of work performed above the critical force to exhaustion and could give an estimative of anaerobic reserve [38], which in this case would be reduced. The importance of anaerobic reserve for water polo is evidenced by high lactate values found in-game (~7.0 mmol∙L^-1^) [1], which sustains the high-intensity efforts during the match [1]. Furthermore, Kontic et al. [44] showed that anaerobic lactate endurance is a significant predictor of offensive and defensive agility for water polo.

This finding regarding the reduction in the anaerobic reserve is different from Bellinger and Minahan [45] who observed an increase in anaerobic capacity after 28 days of β-alanine supplementation measured by a metabolic method on a cycle ergometer. In fact, since acidosis impairs the functioning of non-oxidative enzymes [24,25], β-alanine supplementation could improve the functioning of enzymes and increase anaerobic capacity, different to the findings of the present study. Additionally, the present study demonstrated no change in [La^-^] at any time, showing that at least glycolytic activity did not deteriorate. Furthermore, the magnitude-based inference analyzes showed that the supposed detrimental effect of β-alanine supplementation on W' is *unclear*, so probably has no substantial effect. Thus, this reduction in W' could be a matter of the modeling, which forced W' to reduce as critical force increased with no changes in peak force, mean force, or integral of force during the test. Therefore, the decrease in W' seems to be a relationship with a fragile interpretation of these parameters in the 3min_ALL-OUT,_ rather than a decrease in the anaerobic activity of athletes. In addition, despite some authors assuming the W' from a critical power model as a valid parameter to estimate the anaerobic work capacity, there are other studies reporting that the W' is not valid for this purpose [46–48].

A possible explanation for the mixed findings may be due to the athletes of the present study being responders and non-responders to the effects of β-alanine supplementation cannot be disregarded. In fact, Baguet et al. [49] found a high range of increases in intramuscular carnosine content (15% to 55%) in individuals that received the same daily dose of β-alanine (4.8 g∙day^-1^). Similarly, Saunders et al. [50] showed increases in carnosine content ranging from 17.13 mmol∙kg^-1^dm to 41.32 mmol∙kg^-1^dm between active individuals after 24 weeks of β-alanine supplementation. Furthermore, Saunders et al. [50] demonstrated that the time to reach maximum carnosine content with β-alanine supplementation is from 4 to 24weeks. Thus, the high range in responses to β-alanine supplementation could have influenced the present results.

A more accurate explanation about this topic could have been given if the present study had measured the intramuscular carnosine content. Indeed, the other limitation of the present study was the lack of pH (blood or intramuscular) measurement and the lack of a metabolic method to estimate the anaerobic capacity of the athletes, such as the maximal accumulated oxygen deficit method. Thus, future studies are encouraged to investigate the effects of β-alanine supplementation on maximal accumulated oxygen deficit.

## Conclusion

Therefore, 4 weeks of β-alanine supplementation presented mixed results in water polo players, indicating that this nutritional strategy may not be effective in improving parameters of the TS_GET_ and 3min_ALL-OUT._

## Supporting information

S1 File DATA SET.xlsx. Individual data set for the tethered swimming graded exercise test and three-minute all-out effort.
(XLSX)
